# The Use of Porcelain

**Published:** 1901-05-15

**Authors:** John J. Green


### THE USE OF PORCELAIN.

BY JOHN J. GREEN, D.D.S.

Read before the Southwestern Dental Society, April 10, 1901.

When I announced my subject as porcelain inlays I intended to write about the technique of porcelain work, bakes, lights, shadows, etc., but after further consideration I concluded to write on the introduction of this branch of work in our general practice. My reason for this—and I thought it not presumptuous on my part—is that there are very few that are introducing this line of work in my immediate locality at present, and I thought it would hold true in other sections.

I have been pursuing this line of work for several years with the intention of introducing it into my practice when I thought it desirable.

About six months ago I began to make preparations for it, and now have it properly installed in my office. I am frank to admit that before I became assured of the success of this work I had been prejudiced by the almost universal condemnation of other dentists equally unfamiliar with the work, and among them men of no mean ability. Another reason for distrusting it was that, as a rule, as soon as a dentist is once prepared to introduce it into his practice it has been and is to some degree the fashion to rush into print and advertise as a cure-all, so to speak, something after the fashion of this:

"Dr. Blank to-day placed in his dental rooms an electric furnace to be used in porcelain work, which method was originated by Dr. Blank and is a new departure which bids fair to become very popular as a substitute for gold fillings and crown work, on account of the unsightly appearance of the latter. The porcelain filling has the natural appearance of the teeth, and is a vast improvement over other fillings. Dr. Blank is the first one in this section to introduce porcelain work, and for the next thirty days he will do this class of work free of charge in order to introduce it. He will be pleased to have all teachers in the schools send children to him for examination and free treatment in case they need it. The idea is scientific, rational, and thoroughly modern. We predict for it a revolutionizing influence in dentistry, and very popular from the beginning."

We all understand the injury such an article would do even the most deserving discovery or invention.

By careful study I have become a convert to the the faith that porcelain work has its place in our practice and that it has come to stay, and like other materials it will work out its own salvation, even if abused by unskilled hands and advertising quacks.

I believe it a duty to ourselves, as well as our patients, to uphold it when it is more satisfactory than other materials. We, as practitioners of that part of the human anatomy chosen for our life's work, should not only aim to preserve, but to artistically beautify the cases coming under our care to the best of our knowledge and ability.

It has been my aim to introduce it only where I am quite certain it will be an artistic improvement over other materials, chiefly in the displacement of unsightly gold in prominent cavities. In every instance it will commend itself to patients. I am pleased to announce that it has more than fulfilled my most sanguine expectations. You need not advertise, as it advertises itself in the delight of your patients. Print advertising injures not only ourselves, but the work also.

And now just a few words in regard to the handling of the porcelain before placing it in the patient's mouth. In all dental porcelain work, whether continuous gum, crown, bridge or filling, it is advantageous to bake twice. Porcelain in fusing shrinks one-sixth its bulk, and by two bakings one is able to gage more accurately the size of the piece. I think it the duty of every beginner to see a good biscuit bake in the laboratory of some skilled porcelain worker, for once seen it will seldom be forgotten. And if you haven't a good biscuit bake, it is needless to go further, for it can not be rectified.

If it is underfused it is apt to shrink too much, and if overfused it will be porous. After fusing to a glaze, if you wish to add to it further you must use a lower fusing body, for it is impossible to fuse with the same or a higher fusing body.

In the baking of these bodies it matters not whether you fuse the high heat Close and Consolidated bodies in two minutes, in a Revelation oil furnace, or whether it is done at a heat that requires six or more minutes, it is just as strong if it has been accurately fused. Care should be taken to have the body dry enough before putting it into the intense heat, or it will be spoiled at once. In my practice I use an electric furnace which requires from fifteen to thirty minutes to bake. It takes a little more time, but the danger of overfusing and spoiling it entirely is not nearly so great.

My furnace body is "Close" for crowns and bridges, and "Consolidated" for fillings. You can order these bodies made up from any of your shadings just as you order your teeth. I usually use facings for the six anterior teeth on both maxillaries, but nothing but the regular body for back teeth, and it is surprising how close your matchings for color will be made up in this way.

Possibly the following table will not be out of order here, and may be of help to any who may be in the beginning of this work.

| | | | | | |
|---|---|---|---|---|---|
| Ash's, high heat fuses at about | | | | | 2600° F. |
| Close, | " | " | " | " | 2600° F. |
| Consolidated | | " | " | " | 2500° F. |
| Ash's, low heat | | " | " | " | 2400° F. |
| Downie's | | " | " | " | 1800° F. |

In conclusion, let me say that you will find that porcelain work has its duty to perform and it has come to stay. Of course, it is not applicable for all cases, but to my mind when properly placed it is excelled by no other material.

